# Psychiatric disorders in women hospitalized at the arrazi psychiatric hospital

**DOI:** 10.1192/j.eurpsy.2023.2019

**Published:** 2023-07-19

**Authors:** H. Berrada, F. Azraf, A. Tounsi, F. Laboudi, A. Ouanass

**Affiliations:** Hôpital psychiatrique arrazi de Salé, Sale, Morocco

## Abstract

**Introduction:**

Women get hospitalized for various serious mental disorders that are gender specific, half of them married with children, the other half single\divorced women stigmatized and marginalized in our society.

**Objectives:**

The aim of this study is to describe mentally ill women admitted into the psychiatric hospital, socio demographically and clinically, highlighting differences, specificities and multiple roles distress deviate with the course of disorder.

**Methods:**

This is a prospective cross-sectional study involving 50 patients admitted to Ar-razi Psychiatric Hospital

**Results:**

The average age is 39years, 41% of them are single, with a low educational level (primary school). 77% of our women are from the urban region, 59% are jobless. 19% of patients in our study have positive family history, 65% of them suffered from schizophrenia. 65% of patients are admitted for schizophrenia, followed by bipolar I disorder 22%, MDD is only represented by 10%. 25% of inpatients deal with a toxic habit, 18,5% abuse nicotine and only one woman have tried quitting. Suicide attempts are closely linked to major depressive episodes of MDD and BID, in patients were already under a combination of antidepressants and anxiolytics for at least 3 months. Psychotic features are observed in most of admitted disorders, 80% in BIP and 64% in MDD. According to HAMILTON-DEPRESSION 62,9% of women are admitted for a severe depressive episode, 30% present anxiety comorbidity and a history of CBT sessions months prior hospitalization.

**Conclusions:**

Shading the light into admitted women in psychiatry and deciphering specific demographic, clinical and therapeutic features may improve the global care system and women’s adherence to treatment and follow up.

**Disclosure of Interest:**

None Declared

